# Functional Role of the RNA-Binding Protein Rbm24a and Its Target *sox2* in Microphthalmia

**DOI:** 10.3390/biomedicines9020100

**Published:** 2021-01-21

**Authors:** Lindy K. Brastrom, C. Anthony Scott, Kai Wang, Diane C. Slusarski

**Affiliations:** 1 Department of Biology, University of Iowa, Iowa City, IA 52245, USA; melinda-brastrom@uiowa.edu; 2 Mercury Data Science, Houston, TX 77098, USA; anthony@mercuryds.com; 3 Department of Biostatistics, University of Iowa, Iowa City, IA 52245, USA; kai-wang@uiowa.edu

**Keywords:** *rbm24a*, *sox2*, post-transcriptional regulation, vision, visual assay, microphthalmia, RNA binding protein, zebrafish

## Abstract

Congenital eye defects represent a large class of disorders affecting roughly 21 million children worldwide. Microphthalmia and anophthalmia are relatively common congenital defects, with approximately 20% of human cases caused by mutations in *SOX2.* Recently, we identified the RNA-binding motif protein 24a (Rbm24a) which binds to and regulates *sox2* in zebrafish and mice. Here we show that morpholino knockdown of *rbm24a* leads to microphthalmia and visual impairment. By utilizing sequential injections, we demonstrate that addition of exogenous *sox2* RNA to *rbm24a*-deplete embryos is sufficient to suppress morphological and visual defects. This research demonstrates a critical role for understanding the post-transcriptional regulation of genes needed for development.

## 1. Introduction

Congenital eye defects represent a large class of disorders affecting roughly 21 million children worldwide [1]. Defects can affect every part of the eye from the retina to the lens, but can also include the eye as a whole. Microphthalmia, a smaller than normal eye(s), and anophthalmia, a lack of an eye(s), are both relatively common congenital defects affecting between 1 in 7000 and 1 in 30,000 live births, respectively [2,3,4,5]. 

Mutations in SRY (sex determining region Y)-box2 *(SOX2*) account for approximately 20% of human anophthalmia cases [6]. As a transcription factor, SOX2, often in cooperation with a partner transcription factor, is responsible for the regulation of many genes and is one of the Yamanaka reprogramming factors that regulates pluripotency [7,8,9]. Thus, understanding the regulation of *SOX2* is crucial. While the transcriptional regulation of *SOX2* has been explored, only recently has the post-transcriptional aspect of its regulation been appreciated [10].

Previously, we identified the RNA-binding protein, RBM24, as a post-transcriptional regulator of *Sox2* [11,12]. Consistent with *SOX2′*s known role in microphthalmia and anophthalmia in human patients, knockdown and knockout *Rbm24*-deficient mice and zebrafish often displayed eye defects including microphthalmia and/or anophthalmia [12,13]. In addition to its role in eye development, *rbm24a* knockdown, mutation, and overexpression are also associated with cardiac defects. Previous inquiries found *Rbm24* mutant mice to be embryonic lethal, dying between E7.5 and E14.5. The embryonic lethality observed is thought to be cardiomyopathy due to numerous cardiovascular malformations, which included ventricular septum defects, reduced trabeculation and compaction, dilated atria and ventricle chambers, thinner atrioventricular endocardial cushions, sarcomere disarray, and fibrosis [12,14,15,16]. Most previous research focused on the cardiac phenotypes associated with *rbm24a* depletion. 

It is challenging to study global factors such as Rbm24a due to their wide-ranging impacts on a multitude of target RNAs. We chose to focus on a single proposed RNA target, *sox2*, to perform more in-depth analyses as to the relationship between Rbm24a and *sox2*. To do so, we analyzed the expression pattern of *rbm24a* and found, consistent with previous studies in zebrafish, *Xenopus*, mice, and chick, its expression in the somites, heart, and lens in a spatiotemporal manner [11,14,17,18,19,20,21,22,23]. To study the role of *rbm24a* in zebrafish development, we performed morpholino knockdown and CRISPR mutagenesis which phenocopied previously reported morphants and mutants [19,20,23]. We next performed sequential injection of *rbm24a* morpholino and exogenous *sox2* RNA to determine the extent to which *sox2* can suppress the *rbm24a*-depleted microphthalmia. We found phenotypic suppression of the *rbm24a*-induced microphthalmia. We also performed a visual assay which tests for light/dark detection on these embryos and found partial visually functional rescue by *sox2* RNA. This work highlights the study of post-transcriptional modification and their target RNAs during eye development. 

## 2. Experimental Section

### 2.1. Animal Care

Zebrafish are maintained in standard conditions under the approval of the University of Iowa Institutional Animal Care and Use Committee (#8071513, 13 August 2018). Embryos are collected from natural spawning and raised between 28 and 30 °C. No more than 50 embryos are kept per 100 mm plate. Embryo plates are cleaned of dead daily and water changes are made as needed.

### 2.2. Microinjection

Embryos at the 1–2 cell stage were injected with either a translation-blocking *rbm24a* morpholino (0.6–1.2, 1–1.3, or 1.7–2 ng), splice-blocking *rbm24a* morpholino, or AltR CRISPR construct (3 nL). The morpholinos were ordered from Gene Tools. *rbm24a* AUG MO sequence: 5′-GCATCCTCACGAAACGCTCAAGTGC-3′. *rbm24a* SB MO sequence: 5′-TTGATATAATCCTCACCTGGCTGCA-3′. The AltR crRNA was ordered from IDT [24]. *rbm24a* AltR crRNA sequence: 5′-GGACUUUCCAGUCUGUCUGUGUUUUAGAGCUAUGCU-3′. RNA for *rbm24a* (100–400 pg), *sox2* (200–300 pg), and EGFP (200–300 pg) was generated from cDNA that was cloned into the pCR8/GW/TOPO vector (*rbm24a*, *sox2*; Invitrogen, Carlsbad, CA, USA) or pCS2 + (EGFP, RZPD) before linearized templates were transcribed using the SP6 Ambion mMessage mMachine kit (Invitrogen, Carlsbad, CA, USA). *rbm24a* and *sox2* RNAs have N-terminal Myc tags. *sox2* RNA lacks a 3′ UTR. Microinjection needles were measured via a capillary tube to ensure dosages fell in the aforementioned ranges. Sequential injections were performed utilizing the same needle between morpholino-injected embryos (morpholino-only and morpholino with RNA) and RNA-injected embryos (RNA-only and morpholino with RNA) to ensure consistent dosage between experimental groups. An app was used to calculate the amount of construct injected (https://play.google.com/store/apps/details?id=com.canthonyscott.microinjectioncalc&hl=en_US). When noted, sequential injections were performed on a single day per set and involved the usage of the same clutch of eggs, needle, and dosage for all embryos injected.

### 2.3. Mutagenesis Detection

Uninjected and injected embryos underwent gDNA extraction at 2–4 dpf. Briefly, 20 µL of 50 mM NaOH per embryo was added before embryos were heated at 95 °C for 15 min. Samples were cooled and neutralized with 1 µL of 1 M Tris pH 8.0 per 10 µL of NaOH. The resulting gDNA underwent PCR amplification before being sequenced and insertion/deletion (indels) detection was performed utilizing Synthego ICE [25]. *rbm24a*-Forward-5′-ATGCATACCACGCAAAAGGAC-3′, *rbm24a*-Reverse-5′-CAGTCTGTCTGTCGGTAATCA-3′.

### 2.4. Automated Startle Response

The automated vision startle response, VIZN, was performed on 5 or 6 days post-fertilization (dpf) larvae as previously described [26]. Phenotypically normal larvae were first tested for the ability to swim by being prodded to ensure touch responsiveness and swimming ability, before being sorted and placed in 48-well plates.

### 2.5. Statistical Analysis

Data in tables were analyzed statistically with Fisher’s exact test implement in R [27]. VIZN assays were statistically analyzed by Mann–Whitney.

## 3. Results

### 3.1. Mutation of rbm24a Leads to Microphthalmia and Cardiomyopathy

**Figure 1:**
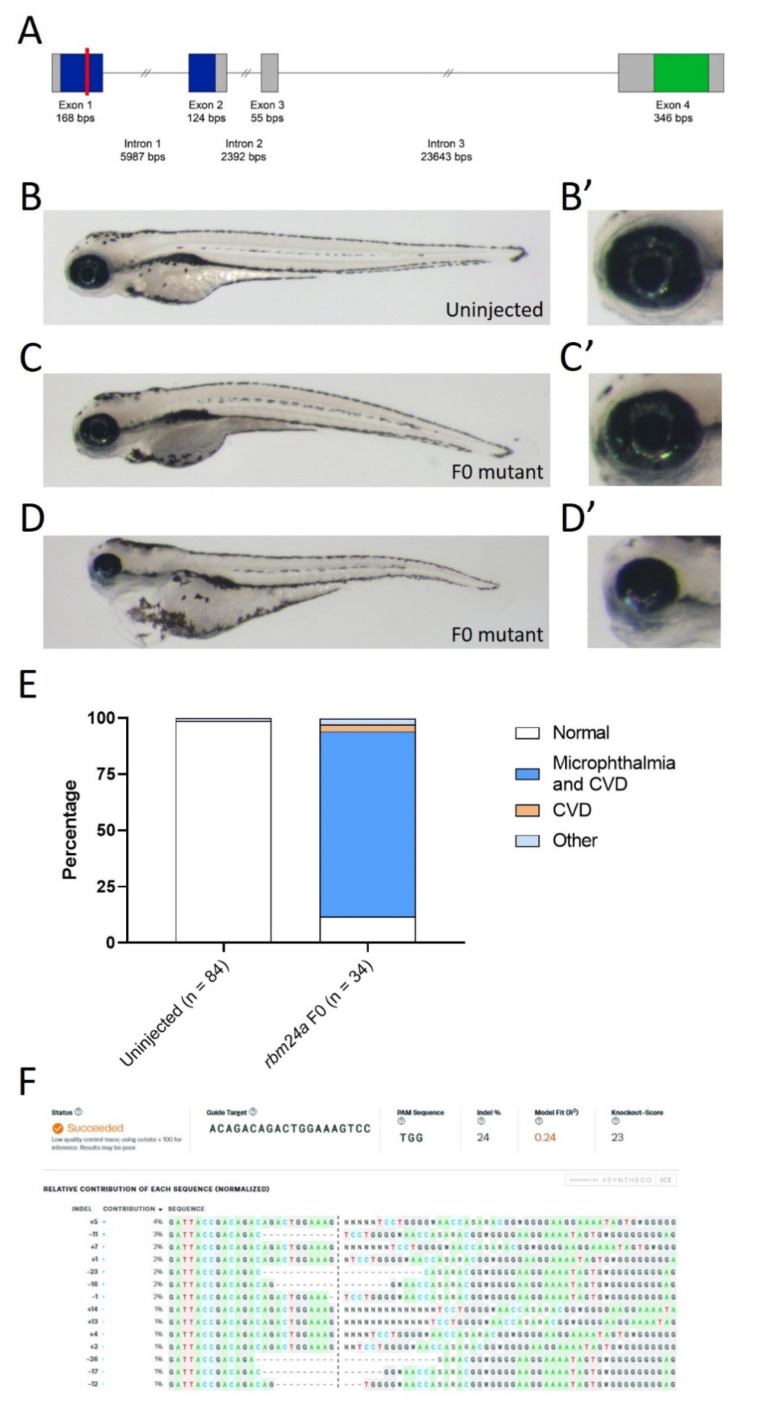
Mutation of rbm24a phenocopies morpholino knockdown. (**A**) Schematic for genetic structure of Rbm24a, with the RNA recognition motif (RRM) in blue and the Ala-rich region in green. The red line indicates the site of the Alt-R CRISPR/Cas0 mutation. (**B**) Uninjected control 4 dpf embryo with wild type morphology. (**B’**) Detail of eye shown in B. (**C**) and (**D**) are Alt-R CRISPR/Cas9-injected embryos showing the variation of the phenotypes. (**C’**) and (**D’**) are detail of eyes found in C and D, respectively. (**E**) Graph of uninjected and CRISPR-injected F0 embryos. (**F**) Synthego ICE analysis of mutations found in F0 mutants. Indels are listed. Images taken at 33×.

Zebrafish *rbm24a* is expressed in the developing heart, somites, and lens [19,20,22]. Translation and splice-blocking morpholino knockdown leads to dose-dependent defects, with lower doses exhibiting microphthalmia, while higher doses display microphthalmia and cardiac defects leading to edema (Supplemental Figure S1). We previously analyzed the microphthalmia phenotype both morphologically and histologically [13]. To validate the usage of the morpholino, we aimed to generate an *rbm24a* mutant. Similar to other organisms, including mice and humans, the zebrafish Rbm24a contains two domains: the RNA recognition motif (RRM) and an Alanine-rich region. The RRM domain spans exons 1 and 2 and is the domain responsible for binding to target RNAs. This domain is highly conserved, with 96.2% identity shared between zebrafish and humans. The Alanine-rich region is in exon 4 and has an unknown function in Rbm24a (Figure 1). Due to the key role of the RRM domain, we designed a CRISPR site in exon 1 which was a predicted null mutation (Figure 1). We performed CRISPR knockout and found phenotypes similar to those of morpholino knockdown. Most CRISPR-injected F0 embryos displayed microphthalmia (small eye(s)) and cardiac edema (fluid around the heart) which is consistent with the expression pattern of *rbm24a*, previously reported morphant phenotypes, and previously reported mutant phenotypes (Figure 1B–E) [19,20,23]. We extracted genomic DNA (gDNA) from both uninjected and CRISPR-injected F0, amplified the region flanking the target cut site, and sequenced the products. We next used Synthego ICE to determine the nature and frequency of indels [25]. Analysis of the CRISPR-injected F0 mutants showed an indel frequency between 20% and 24% when compared to the uninjected control embryos (Figure 1F). There is a statistically significant difference between the phenotypes of uninjected control and CRISPR-injected F0 embryos (*p* < 1 × 10^−16^) (Table 1). 

### 3.2. rbm24a RNA Suppression of rbm24a Morpholino Knockdown 

**Figure 2:**
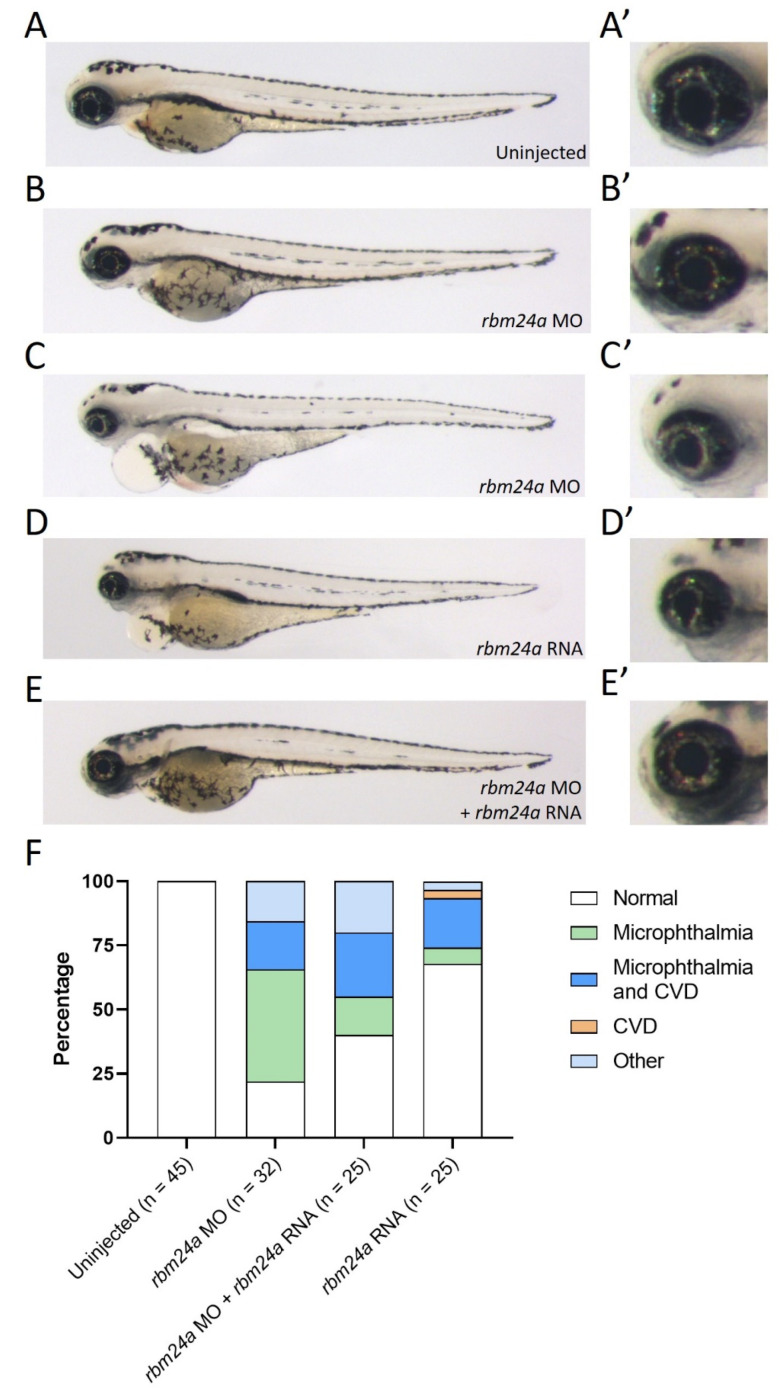
Suppression of *rbm24a* morpholino knockdown with *rbm24a* RNA. (**A**) Uninjected 4 dpf embryo with wild type morphology, (**A’**) detail of eye in A. (**B**) Knockdown of *rbm24a* at low doses leads to microphthalmia, (**B’**) detail of eye in B, while (**C**) is a higher dose showing microphthalmia with cardiac edema, (**C’**) detail of eye in C. (**D**) Injection of *rbm24a* RNA yields phenotypes similar to higher dose knockdown, (**D’**) detail of eye in D. (**E**) Sequential injection of *rbm24a* morpholino and *rbm24a* RNA suppresses the phenotypes, (**E’**) detail of eye in E. (**F**) Graph of phenotypes. Images taken at 33×.

Due to the high number of CRISPR-injected F0 embryos exhibiting both microphthalmia and cardiac edema, we chose to utilize a low dose of translation-blocking morpholino in order to generate microphthalmia-only *rbm24a* knockdown phenotypes. When compared to uninjected embryos, morphant embryos injected with a low dose (1–1.3 ng) displayed only microphthalmia, while embryos injected at a higher dose (1.7–2 ng) phenocopied the CRISPR-injected F0 embryos with both microphthalmia and cardiac edema (Figure 2A–C’). 

As an additional confirmation of the *rbm24a* knockdown phenotype, we next determined the extent to which exogenous *rbm24a* RNA can suppress the knockdown defects. We modified the *rbm24a* RNA construct so the morpholino could not bind to this exogenous *rbm24a* RNA. When injected alone, *rbm24a* RNA led to an overexpression phenotype very similar to the *rbm24a* knockdown phenotype (100–400 pg; Figure 2D–D’). The similarity of the *rbm24a* knockdown and overexpression phenotypes poses a challenge for phenotypic suppression studies, yet when we performed sequential injections of *rbm24a* morpholino and *rbm24a* RNA, we demonstrated phenotypic suppression (Figure 2E–E’). In morpholino knockdown alone, approximately 20% of the embryos were phenotypically normal, while the remaining 80% of embryos displayed defects. When embryos were sequentially injected with *rbm24a* morpholino and *rbm24a* RNA, the number with normal morphology doubled to nearly 40%, resulting in partial suppression of the knockdown microphthalmia defect (*p* = 0.0745) (Figure 2F, Table 2). The overlapping phenotypes generated by two different morpholinos, the suppression of the knockdown phenotype by exogenous *rbm24a* RNA, and the phenotypic similarity between the CRISPR-injected and morphant embryos indicate that the phenotypes observed are specific to *rbm24a*.

### 3.3. EGFP RNA Does Not Suppress rbm24a Morpholino Knockdown Phenotypes 

**Figure 3:**
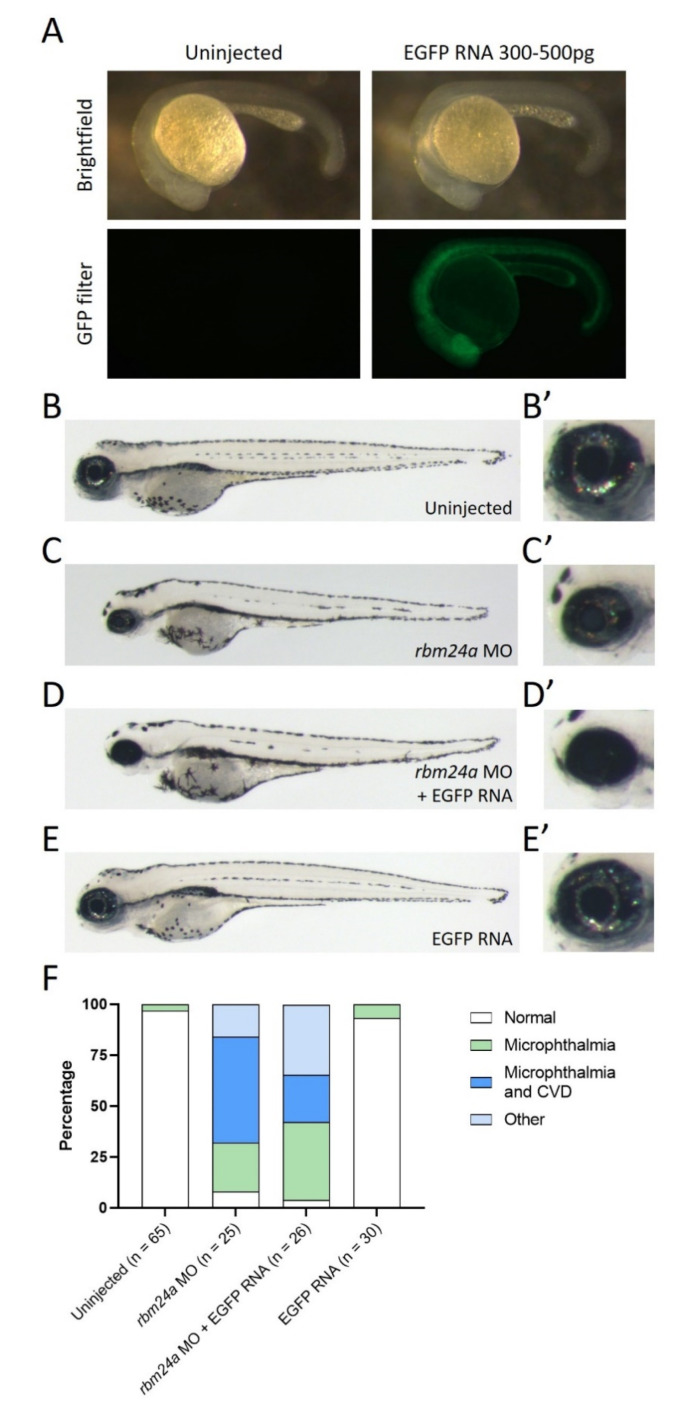
EGFP RNA does not suppress *rbm24a* knockdown. (**A**) Uninjected and EGFP RNA-injected 1 dpf embryos are shown in both brightfield (top) and with a GFP filter (bottom). (**B**) Uninjected 4 dpf embryo with wild type morphology, (**B’**) detail of eye in B. (**C**) *rbm24a* knockdown embryos display microphthalmia with cardiac edema, (**C’**) detail of eye in C. (**D**) Sequential injection of *rbm24a* morpholino and EGFP RNA yields phenotypes similar to knockdown alone, (**D’**) detail of eye in D. (**E**) EGFP RNA-injected embryos are morphologically wild type, (**E’**) detail of eye in E. (**F**) Graph of phenotypes. Image A taken at 62×. Images B-E’ taken at 33×.

Rbm24a is an RNA-binding protein. Therefore, we sought to determine if any exogenous RNA was sufficient to suppress the *rbm24a* knockdown phenotype. We utilized EGFP RNA as a control for RNA injection. EGFP is not naturally found in zebrafish and should have no function. Additionally, we can check for successful injection by screening embryos for EGFP fluorescence (Figure 3A). When compared to uninjected control embryos, both embryos injected with *rbm24a* morpholino (1.7–2 ng) and embryos injected with both *rbm24a* morpholino and EGFP RNA (200–300 pg) showed similar phenotypes, including many with microphthalmia and microphthalmia with cardiac edema (Figure 3B–D’). Injection of EGFP RNA alone yielded a phenotype similar to the uninjected control group (Figure 3E–E’). The number of normal embryos in both the uninjected group and EGFP RNA group is almost identical (*p* = 0.1050), while the number and nature of the affected embryos in both the *rbm24a* knockdown and *rbm24a* knockdown with EGFP RNA are also very similar (*p* = 0.5884) (Figure 3F, Table 3). Taken together, these data suggest that *rbm24a* knockdown defects cannot be suppressed by sequential injection of an unrelated exogenous RNA.

### 3.4. sox2 RNA Phenotypically Suppresses rbm24a Morpholino-Induced Microphthalmia 

**Figure 4:**
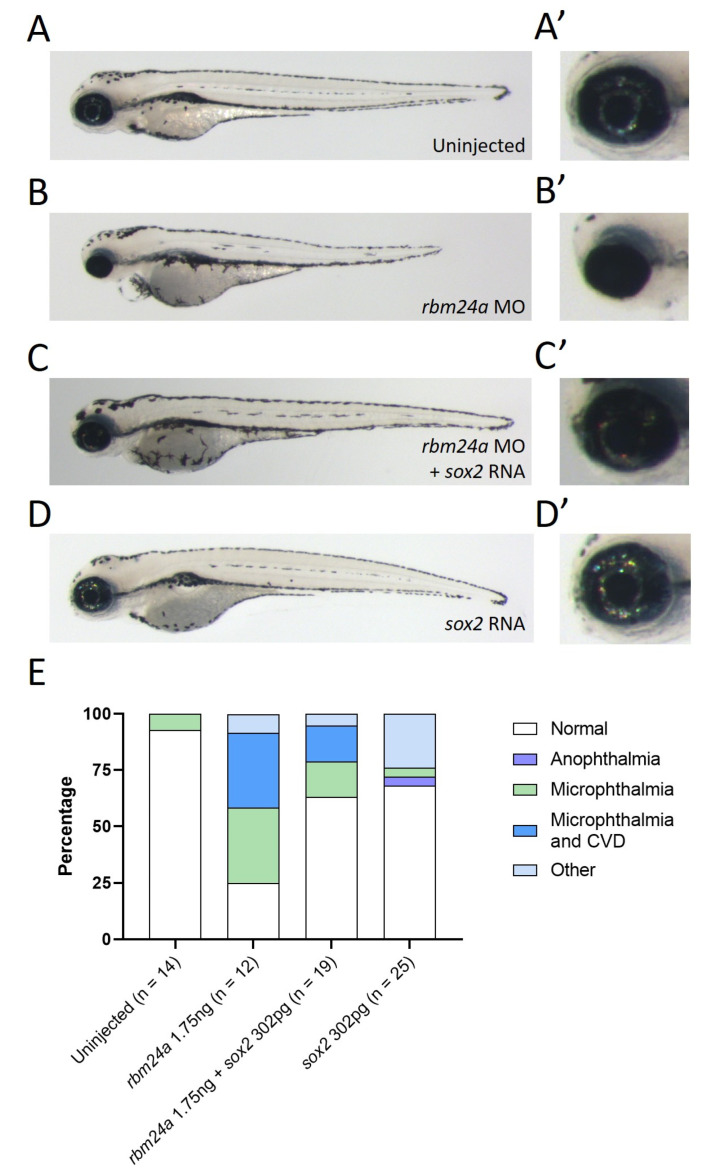
Exogenous *sox2* can suppress *rbm24a*-associated microphthalmia. (**A**) Uninjected 4 dpf embryo with wild type morphology, (**A’**) detail of eye in A. (**B**) Knockdown of *rbm24a* at low doses leads to microphthalmia, (**B’**) detail of eye in B. (**C**) Sequential injection of *rbm24a* morpholino and *sox2* RNA results in a phenotype more similar to wild type than *rbm24a* morphant, (**C’**) detail of eye in C. (**D**) Injection of sox2 RNA alone results in wild type morphology, (**D’**) detail of eye in D. (**E**) Graph of phenotypes. Images taken at 33×.

We previously identified *sox2* as a target of Rbm24a in both zebrafish and mouse models [12]. In that study, we demonstrated that loss of *rbm24a* led to decreased levels of *sox2*. We hypothesize that supplying exogenous *sox2* RNA would supplement the reduced endogenous *sox2* RNA levels. We previously identified the binding site in the 3′ UTR of *Sox2* for RBM24 via mouse cell culture studies [12]. Thus, we generated a zebrafish *sox2* RNA construct which lacked the 3′ UTR to prevent Rbm24a binding (referred to as *sox2* RNA). To test the functional role of *sox2* as a target of Rbm24a, we performed sequential injection of *rbm24a* morpholino followed by *sox2* RNA. Knockdown of *rbm24a* (1.7–2 ng) generated embryos with microphthalmia or microphthalmia with cardiac edema when compared against the uninjected control (Figure 4A–B’). 

In contrast, embryos sequentially injected with both *rbm24a* morpholino and *sox2* RNA (200–300 pg) were often normal in morphology (Figure 4C–C’). Injection of *sox2* RNA alone also generated mostly phenotypically normal embryos (Figure 4D–D’). A majority of uninjected, *rbm24a* morpholino and *sox2* RNA-injected, and *sox2* RNA-injected embryos were morphologically normal with some exceptions in both injection groups. The *rbm24a* knockdown embryos displayed more embryos with microphthalmia or microphthalmia with cardiac edema when compared to the *rbm24a* knockdown with *sox2* RNA, indicating that injection of exogenous *sox2* RNA can partially suppress the *rbm24a* knockdown phenotype (Figure 4E, Table 4). 

### 3.5. EGFP RNA Does Not Functionally Suppress rbm24a-Induced Visual Defects 

**Figure 5:**
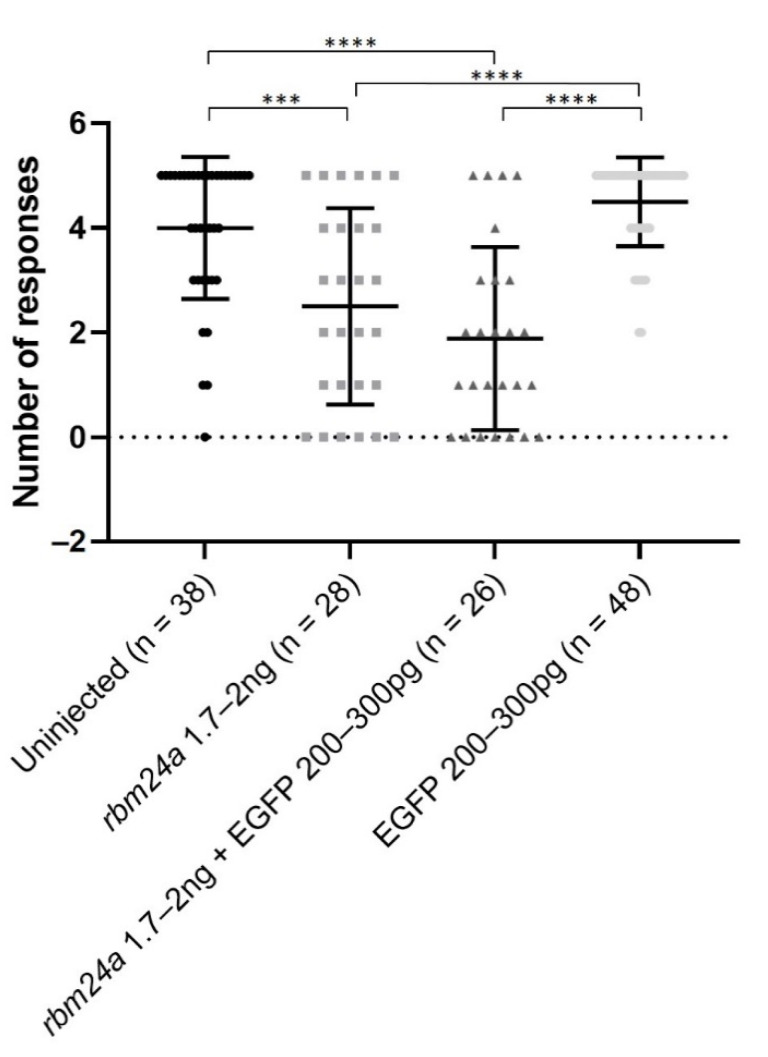
EGFP RNA does not improve visual function of *rbm24a* knockdown embryos. Automated startle response assay (VIZN) was performed on uninjected, *rbm24a* knockdown, *rbm24a* knockdown with EGFP RNA, and EGFP RNA larvae. Knockdown of *rbm24a* inhibits visual function, which was statistically significant compared to uninjected. Addition of EGFP RNA to *rbm24a* knockdown does not statistically significantly increase visual function when compared to *rbm24a* knockdown alone. Injection of EGFP RNA alone does not statistically significantly alter visual function from uninjected. Mann–Whitney *** *p* < 0.001, **** *p* < 0.0001. Nonsignificant interactions not shown.

We next wanted to determine the functional role of *rbm24a* in vision. Previously, we demonstrated that morpholino knockdown of *rbm24a* impacts the startle response in a dose-dependent manner [13]. When 5 to 6 days post-fertilization zebrafish larvae are exposed to an interruption of a constant light source, they perform a characteristic escape response which can be tracked with motion detection cameras and software. We utilized the automated startle response assay (VIZN) which generates five interruptions in light and records the zebrafish movement in response to the interruption in light [26]. Uninjected control larvae responded roughly four out of five times, indicating that they are visually responsive to the stark change in light in the startle response assay. Our previous studies indicated that low-dose knockdown of *rbm24a* maintained light detection [13]. For this study, we utilized a mid-range dose of *rbm24a* morpholino to better evaluate the effectiveness of RNA suppression. For all of our vision assays, we only utilized larvae that were touch responsive with a characteristic swimming response and had no cardiac edema. These *rbm24a* morphant larvae responded to the automated startle response assay (VIZN) significantly fewer times (approximately two out of five times) than the uninjected control (Figure 5). When sequentially injected with both *rbm24a* morpholino and EGFP RNA, larvae had a similar response to the *rbm24a* morpholino-alone larvae and responded about two out of five times. The difference between the *rbm24a* morpholino-only and *rbm24a* morpholino with EGFP RNA was not significantly different. The larvae injected with only EGFP RNA responded roughly four out of five times. There was no significant difference between the uninjected control and EGFP RNA-alone groups (Figure 5).

### 3.6. sox2 RNA Partially Functionally Suppresses rbm24a-Induced Visual Defects 

**Figure 6:**
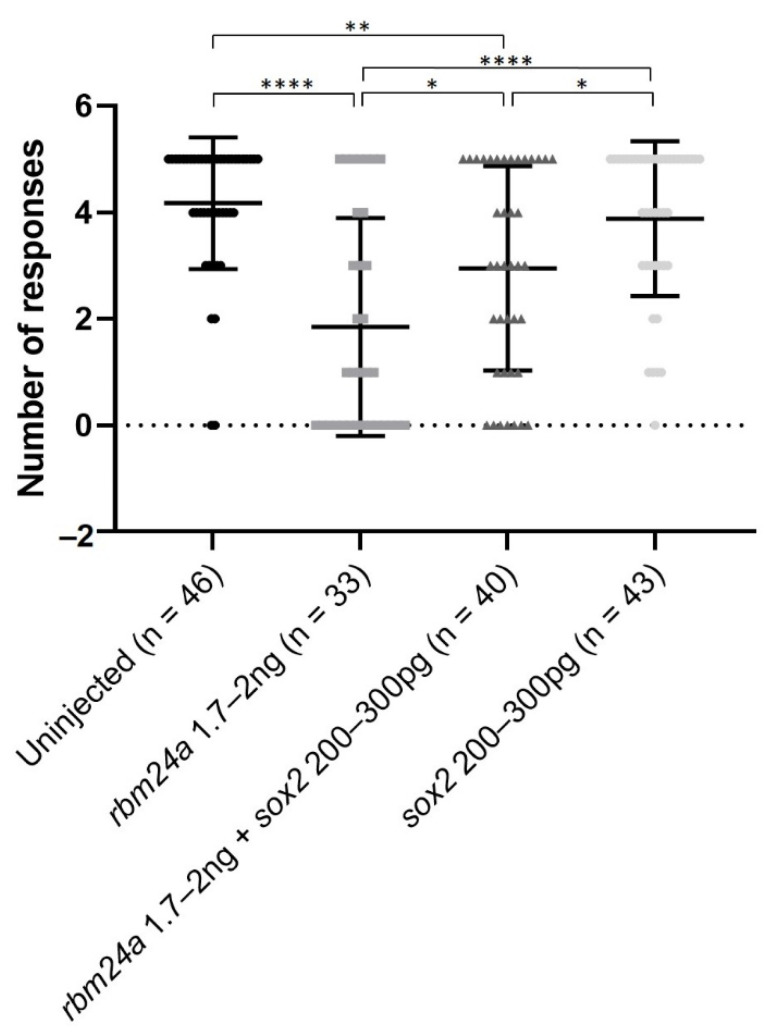
Injection of *sox2* RNA can partially restore visual function by VIZN to *rbm24a* knockdown embryos. VIZN was performed on uninjected, *rbm24a* knockdown, *rbm24a* knockdown with *sox2* RNA, and *sox2* RNA larvae. Knockdown of *rbm24a* statistically significantly inhibits visual function when compared to uninjected. Addition of *sox2* RNA to *rbm24a* knockdown statistically significantly improves visual function when compared to *rbm24a* knockdown, but not to the same level as uninjected. Injection of *sox2* RNA alone does not statistically significantly alter visual function from uninjected. Mann–Whitney * *p* < 0.05, ** *p* < 0.01, **** *p* < 0.0001. Nonsignificant interactions not shown.

We next investigated the extent to which *sox2* RNA can restore visual functionality to *rbm24a* knockdown larvae. We performed VIZN on the uninjected control and *rbm24a* knockdown larvae. Uninjected control larvae responded approximately four out of five times, indicating that they are visually responsive to the startle response assay (Figure 6). As stated previously, we purposely utilized a dose of *rbm24a* morpholino which resulted in larvae responding statistically significantly fewer times (roughly two out of five) than the uninjected control. Sequential injection of *rbm24a* morpholino and *sox2* RNA larvae responded significantly more times (about three out of five) than *rbm24a* morpholino-injected larvae. However, the *rbm24a* morpholino and *sox2* RNA larvae were also statistically significantly different from the uninjected control larvae. Larvae injected with only *sox2* RNA responded similarly (about four out of five times) to the uninjected control (Figure 6). Taken together, these data indicate *sox2* partially restores visual function in *rbm24a* morpholino knockdown larvae.

## 4. Discussion

**Figure 7:**
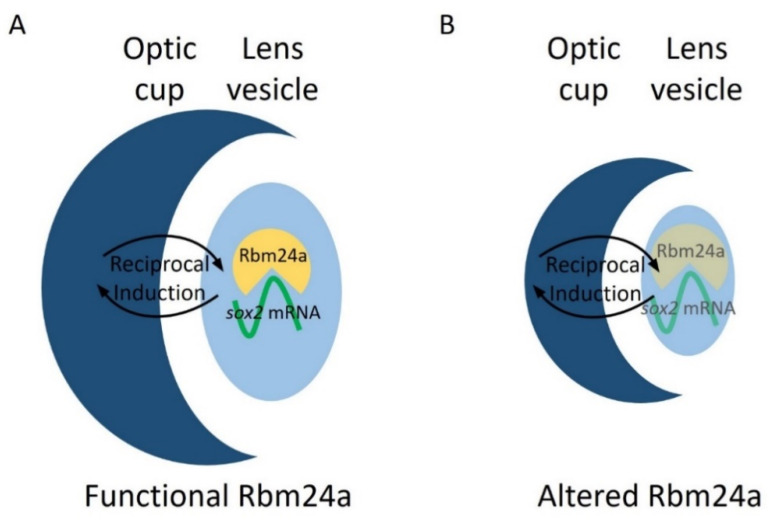
Model for Rbm24a in zebrafish eye development. (**A**) Functional Rbm24a is found in the developing lens of zebrafish. There, the protein acts to bind to and stabilize *sox2* mRNA. This interaction leads to the development of a normal sized lens and, with reciprocal induction signaling between the lens and retina, the retina also develops normally to size-match the lens. (B) With either knockdown or mutation of *rbm24a*, there is lessened Rbm24a protein in the lens which in turn cannot stabilize as many *sox2* mRNA molecules (represented as transparent shapes). The lack of the correct amount of *sox2*, a proliferative factor, causes the lens to develop smaller than normal. However, reciprocal induction has not been affected, which leads to the lens and retina developing small in tandem.

Our previous research indicated *sox2* as a target for Rbm24a and suggests that Rbm24a binds to and stabilizes the *sox2* mRNA transcript [12]. Localization data indicate that *rbm24a* is expressed exclusively in the lens. This suggests that Rbm24a binds to and regulates lens-expressed *sox2* in the lens [17,18,19,20,21,28]. When knocked down via morpholino or mutated via CRISPR, the levels of Rbm24a are depleted which leads to a decrease in the stability and amount of *sox2* mRNA. One possibility is that the reduced levels of *sox2*, a proliferative factor, lead to decreased lens vesicle size. Due to coordinated development between the lens vesicle and optic cup via reciprocal induction, the optic cup develops in tandem with the lens vesicle and results in microphthalmia (Figure 7).

Recently, a zebrafish TALEN mutant for *rbm24a* has been published which phenocopies both our morpholino knockdown and CRISPR-injected F0 defects [23]. In the Shao et al. 2020 study, the authors suggest that microphthalmia is the result of a cardiac morphological defect leading to poor blood flow to the eye. We hypothesized that the microphthalmia was due to target RNA disruption by decreased *rbm24a* expression. In our previously published studies, we identified that RBM24 binds to *Sox2* in mouse cell cultures [12]. From this, we hypothesized that knockdown of *rbm24a* with the addition of exogenous *sox2* would suppress the microphthalmia phenotype. We found that sequential knockdown of *rbm24a* and the addition of exogenous *sox2* RNA generated a phenotype more similar to that of uninjected control embryos than *rbm24a* knockdown (Figure 4A–C’). Our data indicate *sox2* is a main contributor to the *rbm24a*-induced microphthalmia. In terms of the previous study by Shao et al., it is possible that the TALEN mutant was not a null mutation which could have allowed for some function in the lens. Additionally, the utilization of heterotypic parabiosis allows for the potential transfer of more than just blood (including hormones, signaling molecules, etc.) between embryos. It is possible that the transfer of these biomolecules could have played a role in suppressing the microphthalmia phenotype [23]. Follow-up studies utilizing an organ-specific driver would resolve this contrast.

We occasionally observed cardiovascular defects in the *sox2* overexpression embryos, perhaps stemming from the ubiquitous overexpression of *sox2*. Ubiquitously expressed exogenous *sox2* may be able to substitute for cardiac members of the SOX family including *Sox6* which has been shown to be a cardiomyocyte regulator in murine cells, *Sox9* which is involved in heart valve development in mice, and *Sox17* which has been shown to be essential for specification of cardiac mesoderm in mouse cell cultures [29,30,31]. In the future, an eye-specific promoter could be utilized to allow for the study of *rbm24a*/*sox2* interactions with specificity to the eye.

In this study, we demonstrated that knockdown and mutation of the RNA-binding motif protein 24a gene, *rbm24a*, leads to microphthalmia at low-dose knockdown and microphthalmia with cardiac edema at increased knockdown doses. Additionally, we demonstrated that the microphthalmia induced by *rbm24a* functionally impacted the visual capabilities of the larvae, as knockdown fish performed worse in a visual behavior study. Previously, we identified *sox2* as a target of Rbm24a [12]. When sequentially injected, exogenous *sox2* RNA is able to phenotypically and partially functionally suppress the *rbm24a* knockdown larvae. This shows functional validation of a suspected target RNA by an RNA-binding protein. This work broadly demonstrates the key role RNA-binding proteins and post-transcriptional modification plays during development. It also highlights that genes not associated with disease, such as *rbm24a*, can impact a well-studied disease-associated gene (*sox2*), making it critical to better understand post-transcriptional regulation and the potential impacts of RNA-binding proteins and their associated target RNAs.

## Figures and Tables

**Table 1:** Number of phenotypes associated with uninjected control and CRISPR-injected F0 embryos.

	Uninjected	*rbm24a* CRISPR F0
Normal	83	4
Microphthalmia		0
Microphthalmia and CVD ^1^		28
CVD		1
Other ^2^	1	1
Total	84	34

^1^ CVD stands for cardiovascular defect. ^2^ Other is a category reserved for phenotypes that do not fall under the above categories. They are frequently trunk defects.

**Table 2:** Number of phenotypes associated with uninjected, *rbm24a* knockdown, *rbm24a* knockdown with *rbm24a* RNA, and *rbm24a* RNA alone.

	Uninjected	*rbm24a* MO	*rbm24a* MO + *rbm24a* RNA	*rbm24a* RNA
Normal	45	7	10	21
Microphthalmia		14	4	2
Microphthalmia and CVD		6	6	6
CVD				1
Other		5	5	1
Total	45	32	25	31

**Table 3:** Number of phenotypes associated with uninjected, *rbm24a* knockdown, *rbm24a* knockdown with EGFP RNA, and EGFP RNA alone.

	Uninjected	*rbm24a* MO	*rbm24a* MO + EGFP RNA	EGFP RNA
Normal	63	2	1	28
Microphthalmia	2	6	10	2
Microphthalmia and CVD		13	6	
CVD				
Other		4	9	
Total	63	25	26	30

**Table 4:** Number of phenotypes associated with uninjected, *rbm24a* knockdown, *rbm24a* knockdown with *sox2* RNA, and *sox2* RNA alone.

	Uninjected	*rbm24a* MO	*rbm24a* MO + *sox2* RNA	*sox2* RNA
Normal	13	3	12	17
Microphthalmia				2
Microphthalmia and CVD		4	3	
CVD				
Other	1	5	4	6
Total	14	12	17	25

## Data Availability

Data sharing not applicable.

## Supplementary Materials

The following are available online at https://www.mdpi.com/2227-9059/9/2/100/s1, Figure S1: Knockdown of *rbm24a* yields dose-dependent phenotypes at 4 days post-fertilization.
