# Supplementary material for: Functional Role of the RNA-Binding Protein Rbm24a and Its Target sox2 in Microphthalmia
Source: Biomedicines. 2021 Jan 21;9(2):100. doi: 10.3390/biomedicines9020100 (PMC7909789; doi:10.3390/biomedicines9020100)
Supplement: Supplementary file 1 [file biomedicines-09-00100-s001.zip › Supplemental v1.docx]

Article

Functional role of the RNA binding protein Rbm24a and its target *sox2* in microphthalmia

Lindy K. Brastrom ^1^, C. Anthony Scott ^2^ and Diane C. Slusarski ^1,^*

^1^ Department of Biology, University of Iowa, Iowa City, IA 52245, USA

^2^ Mercury Data Science, Houston, TX 77098, USA

**Content:**

**Supplementary Figure 1.** Dose-depended phenotypes of *rbm24a* morpholino.


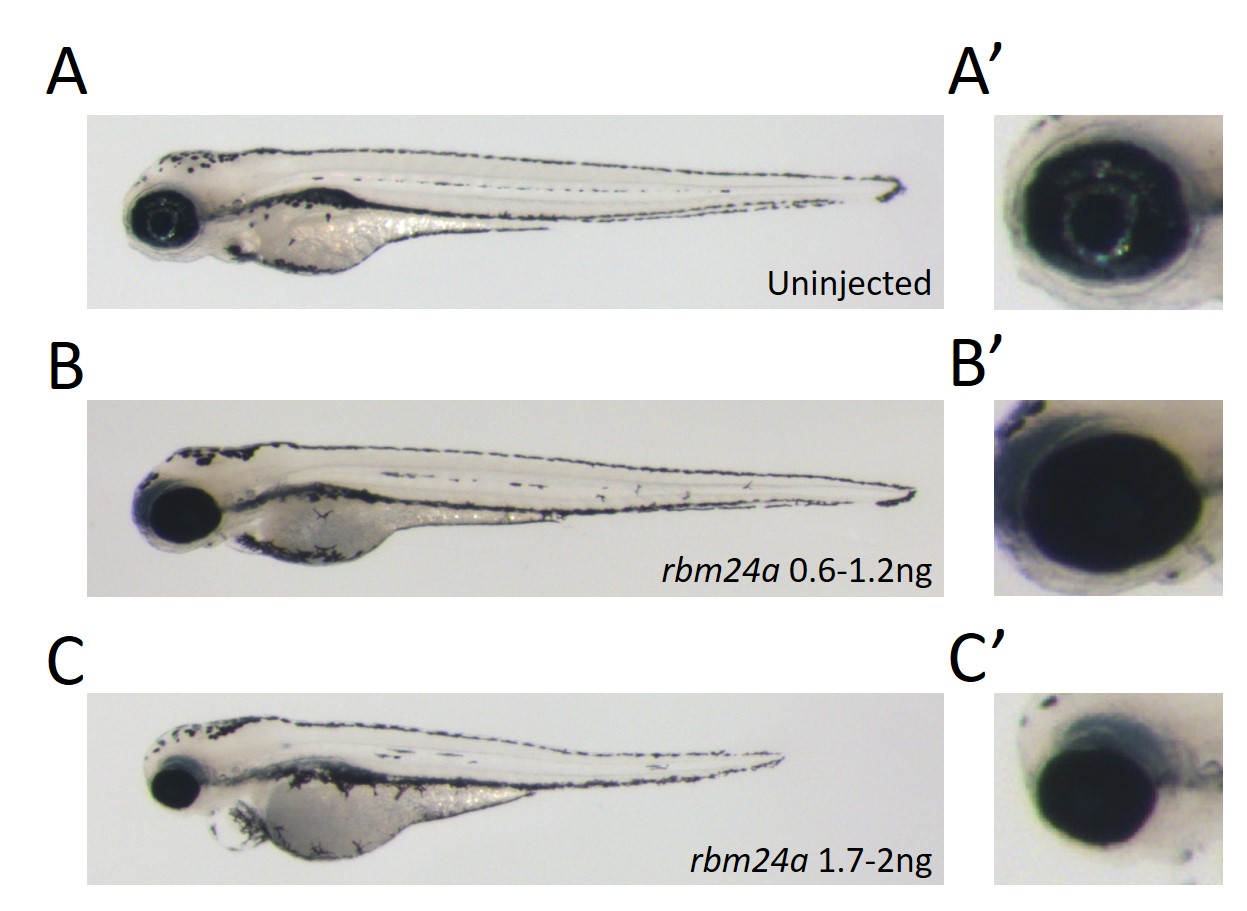


**Figure S1.** Knockdown of *rbm24a* yields dose-dependent phenotypes at 4 days post fertilization. (**A**) Uninjected control displaying wild type morphology. (**A’**) Detail of the wild type eye morphology of A. (**B**) Low dose knockdown of *rbm24a* (0.6-1.2ng) gives subtle microphthalmia. (**B’**) Detail of microphthalmia in B. (**C**) Higher dose knockdown of *rbm24a* (1.7-2ng) embryos display more severe microphthalmia and cardiac edema. (**C’**) Detail of microphthalmia in C.
